# The international anorectal physiology working group (IAPWG) recommendations: Standardized testing protocol and the London classification for disorders of anorectal function

**DOI:** 10.1111/nmo.13679

**Published:** 2019-08-12

**Authors:** Emma V. Carrington, Henriette Heinrich, Charles H. Knowles, Mark Fox, Satish Rao, Donato F. Altomare, Adil E. Bharucha, Rebecca Burgell, William D. Chey, Guiseppe Chiarioni, Philip Dinning, Anton Emmanuel, Ridzuan Farouk, Richelle J. F. Felt‐Bersma, Kee Wook Jung, Anthony Lembo, Allison Malcolm, Ravinder K. Mittal, Franҫois Mion, Seung‐Jae Myung, P. Ronan O’Connell, Christian Pehl, Jose María Remes‐Troche, R. Matthew Reveille, Carolynne J. Vaizey, Veronique Vitton, William E. Whitehead, Reuben K. Wong, S. Mark Scott, All members of the International Anorectal Physiology Working Group

**Affiliations:** ^1^ Queen Mary University of London London UK; ^2^ University of Zürich Zürich Switzerland; ^3^ Medical College of Georgia Augusta Georgia USA; ^4^ University Aldo Moro of Bari Bari Italy; ^5^ Mayo Clinic Rochester Minnesota USA; ^6^ Monash University and Alfred Health Melbourne Victoria Australia; ^7^ Michigan Medicine Ann Arbor Michigan USA; ^8^ AOUI Verona Verona Italy; ^9^ Flinders University Adelaide South Australia Australia; ^10^ University College London London UK; ^11^ National University Hospital Singapore Singapore City Singapore; ^12^ UMC Amsterdam Amsterdam The Netherlands; ^13^ Asan Medical Center Seoul Korea; ^14^ Harvard Medical School St, Boston Massachusetts USA; ^15^ University of Sydney and Royal North Shore Hospital Sydney New South Wales Australia; ^16^ University of California Berkeley California USA; ^17^ Université de Lyon et Hospices Civils de Lyon Lyon France; ^18^ University College Dublin Dublin Ireland; ^19^ Krankenhaus Vilsbiburg and Technical University Munich Munich Germany; ^20^ University of Veracruz Veracruz Mexico; ^21^ University of Colorado Denver Colorado USA; ^22^ St Mark’s Hospital and Imperial College London London UK; ^23^ AP‐HM ‐ Aix‐Marseille University Marseille France; ^24^ University of North Carolina at Chapel Hill Chapel Hill North Carolina USA

**Keywords:** anorectal function testing, anorectal manometry, balloon expulsion test, functional anorectal disorders, rectal sensory test

## Abstract

**Background:**

This manuscript summarizes consensus reached by the International Anorectal Physiology Working Group (IAPWG) for the performance, terminology used, and interpretation of anorectal function testing including anorectal manometry (focused on high‐resolution manometry), the rectal sensory test, and the balloon expulsion test. Based on these measurements, a classification system for disorders of anorectal function is proposed.

**Methods:**

Twenty‐nine working group members (clinicians/academics in the field of gastroenterology, coloproctology, and gastrointestinal physiology) were invited to six face‐to‐face and three remote meetings to derive consensus between 2014 and 2018.

**Key recommendations:**

The IAPWG protocol for the performance of anorectal function testing recommends a standardized sequence of maneuvers to test rectoanal reflexes, anal tone and contractility, rectoanal coordination, and rectal sensation. Major findings not seen in healthy controls defined by the classification are as follows: rectoanal areflexia, anal hypotension and hypocontractility, rectal hyposensitivity, and hypersensitivity. Minor and inconclusive findings that can be present in health and require additional information prior to diagnosis include anal hypertension and dyssynergia.

**Conclusions and Inferences:**

This framework introduces the IAPWG protocol and the London classification for disorders of anorectal function based on objective physiological measurement. The use of a common language to describe results of diagnostic tests, standard operating procedures, and a consensus classification system is designed to bring much‐needed standardization to these techniques.

## Graphical abstract

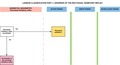

**Abbreviations**
ARM: Anorectal manometry
BET: Balloon expulsion test
C1: Level 1 consensus
C2: Level 2 consensus
C3: Level 3 consensus
DDV: Defecatory desire volume
FCSV: First constant sensation volume
HR‐ARM: High‐resolution anorectal manometry
IAPWG: International Anorectal Physiology Working Group
LLN: Lower limit of normal
MTV: Maximum tolerated volume
RAIR: Rectoanal inhibitory reflex
RST: Rectal sensory test
ULN: Upper limit of normal

**Key Points**

- Anorectal function tests (comprising anorectal manometry, the rectal sensory test, and the balloon expulsion test) are commonly used to evaluate patients with symptoms of anorectal dysfunction; however, practice varies significantly.
- This document summarizes consensus reached by the International Anorectal Physiology Working Group (IAPWG) for the performance of anorectal function testing and introduces a consensus classification for disorders of anorectal function based on objective, physiological measurement.
- The four‐part London classification addresses (a) disorder of the rectoanal inhibitory reflex; (b) disorders of anal tone and contractility; (c) disorders of rectoanal coordination; and (d) disorders of rectal sensation. Findings are defined as major, minor, or inconclusive.

## 1 INTRODUCTION

Symptoms of anorectal dysfunction, characterized by fecal incontinence and/or constipation/evacuation disorders, affect the quality of life of between 1% and 5% of the population.^1^ Anorectal manometry (ARM), the rectal sensory test (RST), and the balloon expulsion test (BET) are the best established investigations for objective assessment of anorectal sensorimotor function, and comprehensive assessment involves a series of measurements that describe voluntary and involuntary control of the anal canal, voluntary and involuntary (reflex) rectoanal coordination, evacuatory function, and rectal sensation.^2^, ^3^, ^4^

Several studies have demonstrated that variations in hardware and protocol (particularly for ARM) influence results of these investigations,^4^, ^5^, ^6^, ^7^, ^8^ and could impact diagnosis and management. Previous position statements and working party reports have already provided guidance on technique for data acquisition, analysis, and reporting.^4^, ^9^, ^10^, ^11^ Despite this, a recent study conducted by our group showed ongoing widespread discordance in practice between institutions.^12^

The introduction of high‐resolution ARM (HR‐ARM) has increased the spatial resolution of data acquisition and provides a continuous visualization of pressure activity from the rectum and the anal canal.^13^ This technique is now used in greater than 50% of institutions performing anorectal physiological tests.^12^ However, this advancement has added a further element of variability in practice, and unless efforts are made early to reach consensus on test performance, this technique may fall victim to the same lack of standardization that has bedeviled other investigations (eg, transit studies) in the field.

The International Anorectal Physiology Working Group (IAPWG) was convened to establish consensus and set minimum standards for the clinical measurement of anorectal function, with a particular focus on HR‐ARM. The following IAPWG consensus guidelines provide a standardized protocol for the performance of anorectal function testing applicable to devices produced by any manufacturer. In addition, the group presents the London classification system for disorders of anorectal function based on objective physiological measurements.

## 2 METHODS

### 2.1 The International Anorectal Physiology Working Group (IAPWG)

The IAPWG is a collaborative of 29 gastroenterologists, coloproctologists, and physiologists from 12 countries (Australia, France, Germany, Ireland, Italy, Mexico, Netherlands, Singapore, South Korea, Switzerland, United Kingdom, and United States) each with an academic interest and clinical practice in anorectal function testing. Six face‐to‐face meetings were held between 2014 and 2018 with each meeting involving at least 12 faculty members and 3 further rounds of remote voting were held between 2016 and 2018, with each round involving all 29 faculty members.

### 2.2 Consensus process

The main objectives of the consensus process were to reach agreement on (i) a minimum standard investigation protocol for HR‐ARM, RST, and BET; (ii) to describe appropriate metrics for describing anorectal function; and (iii) to develop a classification for the interpretation of test results to facilitate more consistent description of pathophysiology.

A combined consensus approach was taken, comprising a Quaker‐based model for face‐to‐face meetings (the principle of which is to reach consensus through discussion, measuring dissent, and achieving unity) and a Delphi method for remote voting (with questionnaires emailed to panel members). Using the latter, consensus for each protocol/terminology statement and each element of the London Classification (titles, decision points, diagnoses, clinical significance, and footnotes) was attained using two rounds of remote voting. Each working group member had the opportunity to choose “agree” (if they agreed with the statement/element as written), “minor concern” (if they agreed with the statement/element in principle but had minor concerns about its description), or “disagree” (if they disagreed with the statement/element as written). To facilitate the binary nature of consensus voting, agree and minor concern votes were amalgamated for counting purposes. Individuals were encouraged to raise points for discussion in a free‐text manner. The results of the Delphi consensus were collated by members of the steering committee (HH, EVC, and SMS) and used to modify statements/elements accordingly prior to rediscussion.

The documented consensus levels represent the final level achieved. The number of participants (>12) and four rounds of written revisions fulfilled the basic criteria required for a guideline decision group (National Institute for Health and Clinical Excellence, April 2007)^14^ and allowed sufficiently reliable estimates at an acceptable cost in terms of travel expenses, etc. The heterogeneity of the group (specialty, nationality, expertise, and equipment used) was deemed desirable to be representative of a range of stakeholders. Agreement was defined without 'weighting' of any participant's views, although some participants contributed more than others to the process. All authors approved the final document.

### 2.3 Levels of consensus

Levels of consensus were defined in advance of voting as follows:

- C1—Level 1 consensus (excellent) defined as > 90% unanimity;
- C2—Level 2 consensus (moderate) defined as 75%‐90% unanimity; and
- C3—Level 3 consensus (none) defined as < 75% unanimity.

## 3 RESULTS AND RECOMMENDATIONS

Recommendations were categorized into (a) study preparation (comprising study indications, patient preparation, digital rectal examination, test specifications); (b) study protocol (comprising study sequence, standard instructions, maneuver definitions, and descriptions); (c) measurements; (d) description of normality; and (e) the London classification. For the protocol and terminology recommendations, the level of consensus (C1, C2, or C3) for each statement discussed has been reported immediately following the respective statement in the text and is shown in Table S1. For the classification system, the level of consensus for each element is shown in Table S2.

### 3.1 Study indications

Anorectal function testing should be performed after referral from a suitable specialist practitioner after organic pathology has been appropriately excluded (C1). Indications may include the following:

- assessment of symptoms of constipation/evacuation disorder (C1)—for identification/quantification of abnormalities of rectoanal coordination, parameters of evacuation and rectal sensitivity (particularly rectal hyposensitivity), and assessment of megarectum/megacolon to exclude hypo/aganglionosis;
- assessment of symptoms of fecal incontinence (C1)—for identification/quantification of impaired anal sphincter function and abnormal rectal sensitivity (both hyper‐ and hyposensitivity);
- assessment of symptoms of functional anorectal pain (C1)—for identification/quantification of anal sphincter hypertension and abnormalities of rectoanal coordination and parameters of evacuation;
- preoperative assessment of anorectal function (C1)—for description of anal sphincter function and parameters of evacuation, particularly if intervention is associated with risks to continence (eg, fistulotomy and lateral sphincterotomy) or ability to evacuate (eg, rectopexy); and
- assessment of anorectal function in patients after obstetric injury/traumatic birth (C1)—if the clinician and patient wish to quantify anal sphincter function prior to future deliveries.

The use of anorectal function testing as a tool for biofeedback was acknowledged by the group; however, guidance was felt beyond the scope of this working party process.

### 3.2 Patient preparation

Prior to the procedure, continuation of all existing medications is acceptable (C1). Patients may eat and drink up until the time of the test (C1). A clinical interview should clarify information such as presenting symptoms, medications and allergies, and pertinent past medical, surgical, and obstetric events. The use of bowel preparation is optional, and patients should be allowed to open their bowels before the procedure should they desire (C1). Preprocedure use of a water or phosphate enema is not contraindicated; however, the use of an enema should be documented to highlight the potential effects on function (C1). Documentation of any medications, particularly those known to affect anorectal function (including analgesics), is recommended (C1).

Prior to commencement of testing, the procedure should be explained, questions answered, and verbal consent obtained. Advice regarding written consent is beyond the remit of these recommendations, and clinicians should follow local or national policy (C1).

### 3.3 Digital rectal examination

Although not expected to be fully diagnostic, a digital rectal examination should be performed before intubation to (a) provide an initial clinical assessment of pelvic floor structure, function, and sensitivity; (b) exclude local pathology and fecal loading (if fecal loading is demonstrated the investigator may consider the use of a tap water or phosphate enema); and (c) check patient understanding of standard instructions such as “squeeze” and “push”.

### 3.4 Test specifications

#### 3.4.1 HR‐ARM

A number of HR‐ARM systems are commercially available.^15^, ^16^ There is little evidence to support one particular configuration over another. Ideally, manometric sensors should record circumferential *not* unidirectional pressure (C1). Recommendations are based on the use of solid‐state systems (C3) but also have relevance to those using water‐perfused HR systems (C2). A minimum longitudinal recording length of 6 cm is required (C1). Thin, flexible catheters are recommended. Rigid, 'high‐definition' catheters may be used, however are not considered standard (C1).

Studies should be performed in the left lateral position (C1) with the hips and knees flexed. To assist probe placement, a non‐anaesthetizing lubricant should be used (C1).

Care should be taken to ensure that the base of any rectal balloon attached to the ARM catheter is sited 3‐5 cm above the upper border of the anal canal, to prevent the balloon impinging upon the anal canal during inflation (C1). The most distal recording sensor should be external to anal verge (C1). If any pain or discomfort is experienced, the probe should be immediately withdrawn. If, on second insertion, there is further discomfort, a medical assessment should be sought.

#### 3.4.2 RST

The test should also be performed in the left lateral position with hips and knees flexed (C1). Studies may be conducted with either an integrated balloon on the manometric probe or with a separate system (C2). Balloon capacity should be no less than 400 mls (as healthy volunteer studies indicate that the upper limit for maximum tolerated volume in health is no greater than 350 mls)^17^, ^18^ (C2) and all components should be latex‐free (C1). Either ramp (continuous) or phasic distension can be used^19^ (C1) though it should be noted that the results derived from each method are not interchangeable. Insufflation should always be performed with air (C1). For ramp distension, the rate should be between 1 and 5 mL/s, and for phasic distension, inflation rate should be set at 10 mL/s (C1).

#### 3.4.3 BET

The balloon expulsion test should ideally be performed using a flexible catheter, up to 16 Fr in diameter, with a non‐latex, compliant balloon attached at the tip (C2). A fixed volume of 50 mL tepid water is recommended for balloon distension (C1), which should be introduced with the subject lying in the left lateral position. To perform the study, the subject should then be transferred to a sitting position, ideally on a toilet in privacy behind a curtain.^20^, ^21^, ^22^ Alternative tests (eg, barium or MR defecography) may be employed to assess parameters of evacuation instead of BET (C1), although it should be noted that diagnostic agreement between these tests is fair at best.^23^ The details of such methods were not discussed in this round of consensus.

## 4 STUDY PROTOCOL

### 4.1 Study sequence

**Figure 1:**
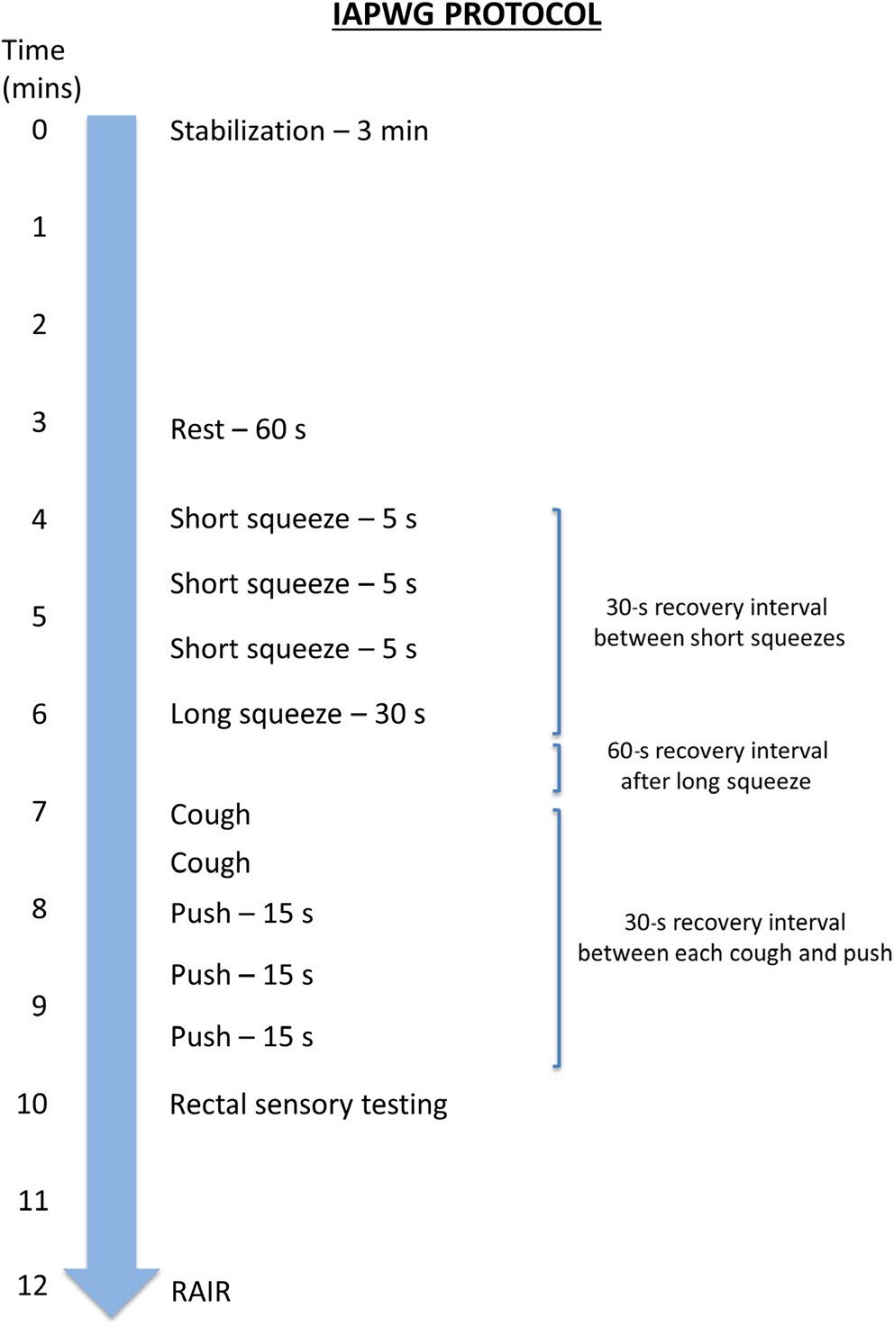
Schematic of the IAPWG standard protocol for high‐resolution anorectal manometry and rectal sensory testing. The balloon expulsion test should be performed either immediately before or after this protocol of anorectal manometry and rectal sensory testing

The agreed standardized protocol for anorectal function testing is outlined in Figure 1, and this scheme as a whole achieved unanimity (C1). Particular attention should be paid to the recommended maneuver durations and to the recovery intervals between maneuvers. The levels of consensus reached for each element of the protocol are outlined in Supplementary Table 1 and are further described in “maneuver descriptions” below. The study time for the IAPWG protocol of HR‐ARM, RST, and BET is expected to be between 15 and 20 minutes, though total time including a clinical assessment will vary between institutions.

**Table 1:** Table describing recommended measurements for high‐resolution anorectal manometry, the balloon expulsion test, and the rectal sensory test

Test	Maneuver	Measurement	Definition	Measurement type		Units	Consensus
				Quantitative	Qualitative		
HR‐ARM	Rest	Anal resting pressure	Mean maximum pressure measured from the whole anal canal over a 60‐second recording period	x		mmHg	C1
		Ultraslow waves	The presence of repeated pressure oscillations within the anal canal, occurring at 1‐2 per min		x	Present/ absent	C1
	Squeeze	Anal squeeze pressure	Maximum incremental pressure observed during the 5‐s short squeeze	x		mmHg	C2
	Long squeeze	Endurance squeeze pressure	The duration of time the subject under study can voluntarily sustain an increase in anal pressure > 50% of maximum incremental squeeze pressure during the 30‐second long squeeze	x		secs	C1
	Push	Rectal pressure change during push	Maximum pressure change recorded within the rectum during the push manoeuver	x		mmHg	C1
		Anal pressure change during push	Maximum pressure change recorded within the anal canal during the push manoeuver	x		mmHg	C1
	Cough	Rectal pressure during cough	Maximum pressure recorded within the rectum during the cough manoeuver	x		mmHg	C1
		Anal pressure during cough	Maximum pressure recorded within the anal canal during the cough manoeuver	x		mmHg	C1
	RAIR	Rectoanal inhibitory reflex	Reflex reduction in maximum anal pressure in response to rapid distension of the rectum		x	Present/ absent^a^	C1
BET		Balloon expulsion time	Time taken in seconds to expel a rectal balloon	x		secs^b^	C1
RST	Rectal sensory thresholds^c^	First sensation volume	The minimum balloon insufflation volume required to elicit a sensory response	x		mls	C1
		Desire to defecate volume	The balloon insufflation volume required to elicit a sustained desire to defecate	x		mls	C1
		Maximum tolerated volume	The balloon insufflation volume that causes an intolerable desire to defecate	x		mls	C1

Abbreviations: HR‐ARM: high‐resolution anorectal manometry, RAIR: rectoanal inhibitory reflex, BET: balloon expulsion test, RST: rectal sensory test.

a The volume required to elicit the RAIR should also be documented.

b The presence or absence of the desire to defecate during the procedure should also be documented.

c Sustained urgency volume threshold is optional and defined as the balloon insufflation volume required to elicit a sense of fecal urgency.

### 4.2 Maneuver descriptions

#### 4.2.1 HR‐ARM

*Stabilization period:* Following catheter insertion and prior to test maneuvers, a 3‐minute period of stabilization should be observed to allow anal tone to return to baseline after intubation (C1).

*Rest:* This is the maneuver that measures basal anal tone at rest. It is measured over 60‐s (C2). During recording, the patient should be reminded to relax and remain quiet to avoid movement artifact (C1). During this maneuver, and during the familiarization period, ultraslow waves (occurring at a frequency of 0.5‐2 cycles per minute) may be seen.

*Squeeze:* This is the maneuver that records the anal pressure during voluntary effort to contract the anus/pelvic floor (C1). Three squeezes are performed during the protocol, each of 5‐second duration separated by a 30‐s between‐maneuver recovery interval. The best of three attempts is used for analysis (C1).

*Long (endurance) squeeze:* This is the maneuver that records the anal pressure during sustained voluntary effort over 30 seconds aiming principally to describe fatigue over time rather than purely contractile ability as measured during 'squeeze' (above). A single endurance squeeze is performed followed by a 60‐s between‐maneuver recovery interval (C1).

*Cough:* This is the maneuver that measures rectoanal pressure changes during cough, that is, assesses the reflex increase in anal sphincter pressure during an abrupt change in intra‐abdominal/intrapelvic pressure. Two coughs are performed separated by a 30‐s between‐maneuver recovery interval. It is important that the practitioner ensures adequate effort during this maneuver and that each cough is a single (not double or multiple) cough. The best attempt (defined as the attempt associated with the greatest increase in rectal pressure) is used for analysis (C1).

*Push:* This is the maneuver that measures anal and rectal pressure changes during simulated defecation. Three pushes are performed during the protocol, each of 15‐s duration (C3) separated by 30‐s between‐maneuver recovery intervals. Rectal balloon insufflation is not mandated during this maneuver (C1). Due to the high rate of false‐positive results related to patient and technical factors,^2^ the best (defined as the most qualitatively normal) of three attempts should be used for analysis (C1).

*RAIR:* This is the procedure which measures reflex anal response to rapid rectal distension. A normal response is characterized by an anal pressure decrease during rectal balloon distension. A single RAIR is performed with a starting volume of at least 30 mls, though it should be noted that failure to elicit the RAIR may be seen with low distending volumes in a large capacity rectum. Therefore, if megarectum is suspected, the test should be repeated with increasing balloon volumes (C1).

#### 4.2.2 RST

*Rectal sensory test:* This is the procedure that assesses rectal sensitivity to distension utilizing a rectal balloon placed at least 3‐5 cm above the upper border of the anal canal (C1). The balloon volume is recorded for each of three patient‐reported sensory thresholds: first constant sensation volume (FCSV), desire to defecate volume (DDV), and maximum tolerated volume (MTV) (C1). A fourth sensory threshold (sustained urgency volume) is optional (C1).

#### 4.2.3 BET

*Balloon expulsion:* This is the procedure that measures ability, based on time taken, for a subject to expel a balloon from the rectum (C1).

### 4.3 Standard instructions

Instruction and verbal feedback have been demonstrated to influence the results of anorectal function testing, particularly HR‐ARM, and therefore, consistency of command is essential.^8^ The following statements have been provided as examples of how to simply describe test components, though it should be noted that significant cultural variation exists and that the exact language used for each instruction was not subject to consensus voting. Patient understanding of the commands should be assessed during clinical examination to prevent suboptimal results. During the study, instructions should be given shortly before each maneuver (C2).

#### 4.3.1 HR‐ARM

*Short squeeze:* “Squeeze as hard as you can for 5‐s as though you are stopping yourself passing wind or stopping yourself opening your bowels.”

*Long (endurance) squeeze:* “Squeeze as hard as you can for as long as you can.” The practitioner should give cues every 5‐s saying “keep squeezing, keep squeezing.”

*Cough:* “Please give a single cough.” The practitioner should demonstrate a cough and emphasize that a single (not double) cough is required.

*Push:* “Push down as though you are sitting on the toilet opening your bowels/ passing a bowel movement/ trying to defaecate.”

#### 4.3.2 RST

“I am going to put some air into the balloon. Tell me when you first feel a sensation inside your bottom that doesn't go away” (first constant sensation volume), “when you feel a constant urge to defaecate/ open your bowels” (desire to defecate volume), “and when it becomes too uncomfortable and you need me to stop” (maximum tolerated volume).

#### 4.3.3 BET

“Try to push the balloon out into the toilet like you're opening your bowels/ passing a bowel movement/ trying to defaecate.”

### 4.4 Measurements

A combination of qualitative and quantitative measurements for describing outcomes is recommended with definitions and units outlined in Table 1.

### 4.5 Description of normality

In this first iteration of recommendations, due to the variability of existing equipment, protocol, and practice, it was felt beyond the remit of the group to recommend specific normal values. However, the description of normality was the subject of significant discussion. The following recommendations were agreed upon:

- if normal values are based on published data, equipment setup and procedure should be identical to that described in the referenced manuscript (C1); and
- if normal values are based on a local study of healthy volunteers, some consideration should be taken to appreciate variability seen in gender (C1), parity (C2), and age (C2).

### 4.6 The London classification of anorectal physiological dysfunction

The following classification was developed to describe findings from the combined results of HR‐ARM, BET, and RST.

**Figure 2:**
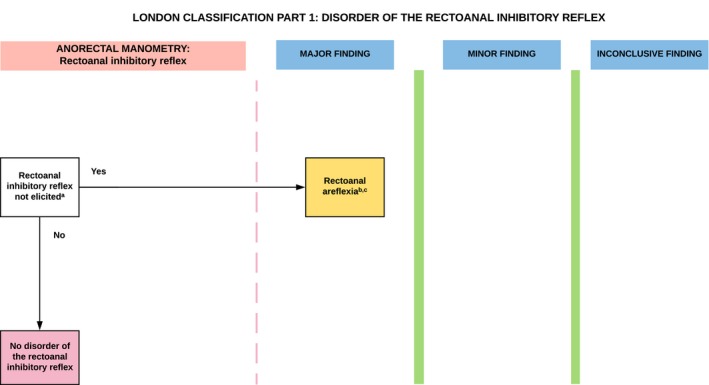
IAPWG classification part 1: Disorder of the rectoanal inhibitory reflex. For this and subsequent figures, the diagrams are color‐coded for clarity: (i) white boxes represent manometric findings or decision points; (ii) yellow boxes represent the resultant diagnosis; and (iii) pink boxes represent a 'negative/normal study'. ^a^Minimum volume required to elicit reflex not established in the literature: failure to elicit a RAIR may be seen with low distending volumes in a large capacity rectum. ^b^RAIR not elicited is a pattern not seen in health but may be found in asymptomatic patients following rectal resection / ileal pouch anal anastamosis, anal hypotonia, faecal loading or megarectum. ^c^May indicate the need for further investigation to exclude aganglionosis expecially in paediatric populations and adult patients with co‐existent megarectum/megacolon. All results to be interpreted in the context of adjunctive testing

**Figure 3:**
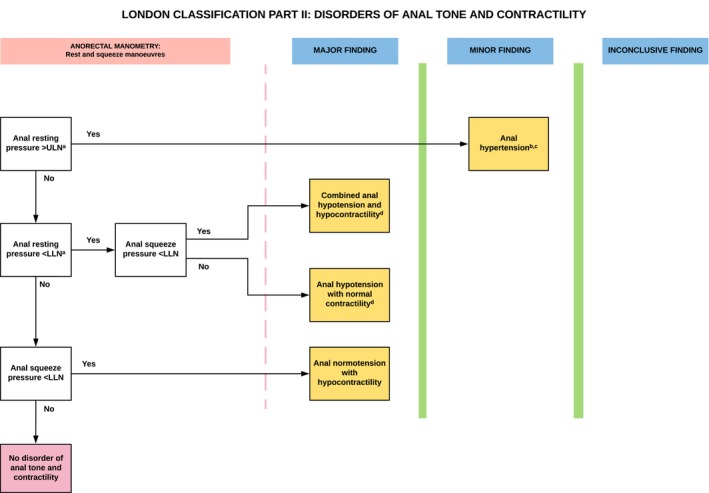
IAPWG classification part 2: Disorders of anal tone and contractility. ^a^The functional anal canal length may be measured, as a short anal canal can be associated with anal hypotonia, but its use as a diagnostic criterion in isolation is unproven. ^b^may be associated with slow and/or ultraslow waves, however the clinical significance of these has not been established. ^c^this finding may have greater clinical significance in certain patient groups (e.g. chronic anal fissure, levator ani syndrome or proctalgia fugax). ^d^addition of an abnormal cough response may indicate a more severe phenotype (whereas preservation may suggest a target for biofeedback) but its use as a diagnostic criterion is unproven. All results to be interpreted in context of adjunctive testing LLN: Lower limit of normal ULN

**Figure 4:**
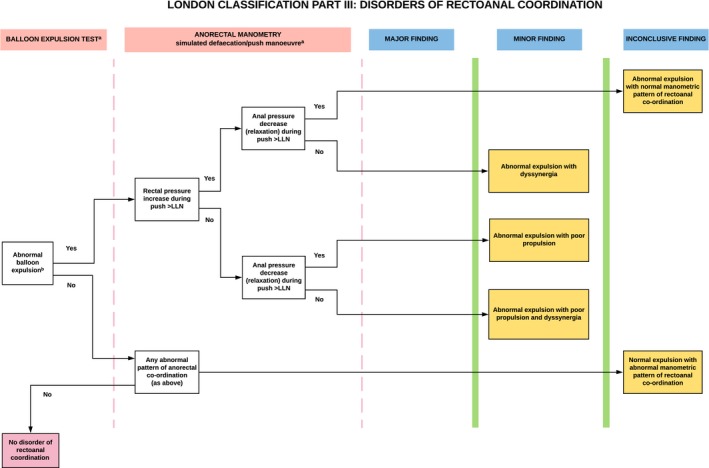
IAPWG classification part 3: Disorders of rectoanal coordination. ^a^requires the use of both balloon expulsion test and anorectal manometry. ^b^or impaired evacuation of contrast medium (prolonged evacuation end time and/or reduced percentage of contrast emptied) on alternative testing e.g. barium or MR defaecography. All results to be interpreted in context of adjunctive testing

**Figure 5:**
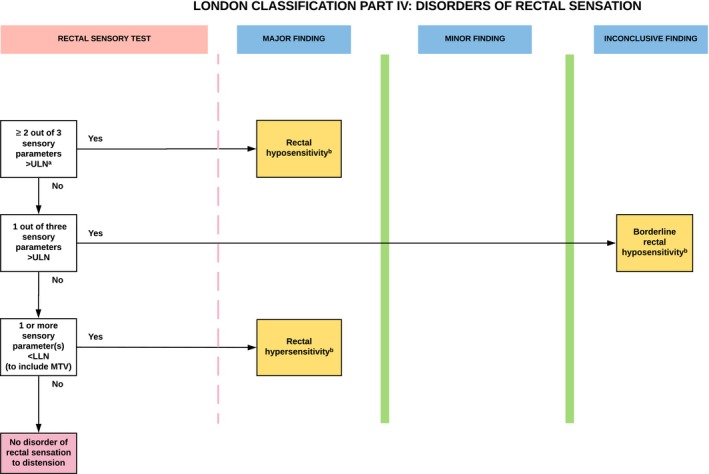
IAPWG classification part 4: Disorders of rectal sensation. ^a^sensory parameters are: first constant sensation volume (FCSV), desire to defecate volume (DDV) and maximum tolerated volume (MTV). ^b^abnormal results may be further described using additional methods (e.g. barostat to assess compliance). All results to be interpreted in context of adjunctive testing

Due to the multicomponent nature of anorectal function testing, it has been divided into four parts and a single study may have an outcome associated with more than one part of the classification:

- Part 1: disorder of the rectoanal inhibitory reflex (Figure 2);
- Part 2: disorders of anal tone and contractility (Figure 3);
- Part 3: disorders of anorectal coordination (Figure 4);
- Part 4: disorders of rectal sensation (Figure 5).

As per the Chicago Classification of esophageal motility disorders,^24^ diagnoses are categorized accordingly:

- a *major finding* is a pattern not seen in control subjects and is likely to represent a physiological alteration associated with symptom generation;
- a *minor finding* is a pattern that is seen in patients with anorectal symptoms, however may also be seen in control subjects and may represent a physiological alteration associated with symptom generation; and
- an *inconclusive finding* is a pattern that is seen in patients with anorectal symptoms, but also seen in control subjects. Such findings may be associated with symptom generation, though the relevance is yet to be fully determined.

It should be noted that the results of two maneuvers (cough and long squeeze), though described in the protocol, do not form part of the diagnostic classification. The consensus group acknowledges the widespread use of these maneuvers but did not feel that inclusion in the classification could be justified at the present time.

For the purposes of uniformity, the diagnostic classification introduces some key terms to describe physiological features of interest: (hypo/hyper)*tension* to describe anal resting tone, (hypo)*contractility* to describe anal squeeze, *expulsion* to describe ability to expel a rectal balloon, *propulsion* to describe generation of an adequate increase in rectal pressure during push, *dyssynergia* to describe failure of coordinated anal relaxation during push, and (hypo/hyper)*sensitivity* to describe rectal sensation.

Levels of agreement for each element of this classification are presented in Table S2.

## 5 DISCUSSION

As patient‐reported symptoms are known to be a poor indicator of underlying pathophysiology,^18^, ^25^ anorectal function testing should be seen as a necessary component of clinical evaluation for patients in whom advanced management strategies are being considered.^2^ The IAPWG recommendations for the performance and interpretation of anorectal manometry (ARM), the rectal sensitivity test (RST), and the balloon expulsion test (BET) mark a major step forward in the field of these investigations and, in particular, provide the first consensus‐based approach to standardization of these investigations in the high‐resolution (HR‐)ARM era. Though little HR‐ARM‐specific guidance is presented, we see this as the necessary bridge to uptake of this technology as a whole. Additionally, the group presents “The London Classification” for the diagnosis of disorders of anorectal function based on objective, physiological measurements. The clinical relevance of findings is indicated by the hierarchical division of findings into (a) major disorders that are not seen in health; (b) minor disorders that may be pathological in patients with symptoms, but that can also be seen in health; and (c) inconclusive findings that may be pathological but that require confirmation by additional testing. This approach follows the model of the Chicago Classification for disorders of esophageal motility and the Lyon Classification for gastroesophageal reflux disease.^24^, ^26^ The IAPWG envisage that, similar to progress made in the upper gastrointestinal tract,^27^ agreement on the performance and analysis of anorectal tests will improve the quality of investigations wherever applied, facilitate results interpretation, collaborative research, and technique development.

This new framework provides a common language with which to describe results of anorectal function testing and as such should be viewed as complimentary to existing disease classifications such as the Rome Classification.^28^ The anorectal disorders section of Rome defines disease entities based on a combination of symptoms and physiological findings but provides limited advice on how to describe abnormal results. The IAPWG protocol and the London Classification provide a standard nomenclature for description of alterations in motor and sensory anorectal function. This working party propose that such a framework be embraced by a future iteration of Rome where both incontinence and constipation/evacuation disorders could be subclassified according to physiological phenotypes. Given the results of recent studies that suggest treatment response differs according to the underlying causes of incontinence^29^ and constipation,^30^ a clear definition of disease phenotypes has the potential to aid treatment stratification and clinical outcomes.

### 5.1 Limitations

The group acknowledge a number of limitations of this consensus document. Firstly, due to the nature of variability in current practice, paucity of data supporting protocol elements, and in particular the recent introduction of HR‐ARM, the vast majority of recommendations reflect coalescence of expert opinion, rather than systematic review of clinical evidence. The reader should therefore bear in mind that what is not normal does not necessarily constitute disease and that outcome studies to assess the clinical utility of this classification for the direction of specific interventions are needed with refinement of the classification system as further data emerge. Nevertheless, we believe that further improvement in practice can only begin from a common starting point, and the presented consensus reflects that sentiment.

Secondly, due to the nature of the task in hand, the consensus describes only 3 simple office‐based tests of function. In particular, as multiple factors are involved in the control of continence and evacuation, the RST and the BET are generally considered as screening tests to be used prior to full assessment with other investigations. Should an abnormality be demonstrated, a comprehensive pelvic floor evaluation may involve more thorough assessment with complimentary investigations of structure or function such as endoanal ultrasound, barium/magnetic resonance defecography, and the rectal barostat which may confirm (or refute) physiological findings such as dyssynergia^31^, ^32^ that are currently labeled as 'inconclusive' by the London Classification. It should be noted however (with particular regard to tests of evacuation) that there is considerable disagreement between the results of available investigations,^23^ and though defecography has the advantage of better describing anatomical *vs.* functional causes of impaired evacuation, the BET in particular is the only test which has been shown to predict the response to biofeedback.^30^

Thirdly, due to the heterogeneity of current data and equipment available, we have not recommended specific, quantitative 'reference limits' for diagnosis of anorectal disorders but, instead, have elected to describe findings in accordance with the upper and lower limits of 'normal'. We acknowledge that female sex, advanced age, and parity are associated with a deleterious effect on sphincter and rectal sensory function and that normal ranges should reflect this. With time, based on the IAPWG protocol, normograms for the physiological variables will be generated as they have been for other biomarkers that vary with age in healthy women and men (eg, bone density^33^).

Finally, although it was the aim of this group to facilitate standardization of *high‐resolution* manometry, all current recommendations can be applied to conventional technology. This was principally because little published data existed to specifically support additional benefits of HR‐ARM. These data are now emerging with recently published evidence, suggesting improved diagnostic accuracy of HR‐ARM,^34^, ^35^ and describing novel functional metrics which may prove worthy of inclusion in further iterations of this classification.^36^ It is clear that publication of the Chicago Classification soon after high‐resolution esophageal manometry 'moved from research into clinical practice' was instrumental in driving its acceptance.^37^ It is only at a later stage that esophageal HRM was shown to have higher interobserver agreement and to increase diagnostic yield and accuracy for motility disorders.^27^, ^38^

### 5.2 Areas for future investigation

In general, an excellent level of agreement was achieved; however, several points failed to reach 90% consensus. This highlighted a number of areas with need for further investigation which include (a) the influence of HR‐ARM software/hardware on results reporting (extrapolation from esophageal HRM suggests this may be important); (b) impact of HR‐ARM with spatiotemporal presentation of pressure data on interobserver agreement; (c) the diagnostic and clinical utility (in terms of yield or disease stratification) of existing and novel HR‐ARM metrics of anorectal function. For instance, the endurance squeeze and cough maneuvers have virtually no evidence to support their use as specific measures of anorectal function (despite, for instance, many working group members reporting the anecdotal utility of cough for describing a more severe phenotype of anal hypocontractility). In addition, previously reported manometric features of anal function (eg, transient anal sphincter relaxations) have been omitted due to time taken to observe these phenomena and the lack of robust evidence of their clinical utility. High‐quality diagnostic accuracy studies^39^ are recommended, (d) re‐evaluation of some currently used methods describing anorectal coordination. In particular, the qualitative description of dyssynergia by the Rao criteria is long‐standing^40^ and has been applied successfully also in HR‐ARM studies^31^, ^41^; however, recent evidence has questioned the usefulness of quantitative metrics (eg, rectoanal pressure gradient) due to significant overlap between HR‐ARM findings in patients with dyssynergia and those in apparently healthy volunteers.^42^ Definitions and clinical significance of HR‐ARM in this area were both major areas of debate in group meetings and should be the focus of further study.

## 6 CONCLUSIONS

The IAPWG protocol incorporating HR‐ARM, RST, and BET together with the London Classification of anorectal disorders provides a much‐needed framework for clinicians performing and interpreting tests of anorectal function. It is expected that these recommendations will evolve as experience with this technology increases and data from physiological and clinical studies emerge.

## CONFLICT OF INTEREST

Donato Altomare, William Chey, Phil Dinning, Anton Emmanuel, Ridzuan Farouk, Richelle Felt‐Bersma, Kee Wook Jung, Anthony Lembo, Malcolm, Seung Jae Myung, and Christian Pehl: None; Adil Bharucha holds patents jointly with Medtronic Inc and Medspira Inc; Rebecca Burgell is a speaker for Bayer and advisory board member for Allergan and Anatara; Emma Carrington is a consultant provided lectures and training courses for Laborie; Guiseppe Chiarioni is a member of the anorectal committee of the Rome Foundation; Mark Fox has received research funding from Covidien/Medtronic and speaker fees from Covidien/Medtronic, Sandhill, Medical Measurement Systems/Laborie, Reckitt Benckiser, and Mui Scientific; Henriette Heinrich is a member of Honorarium for teaching from Laborie; Charles Knowles is a consultant and invited speaker provided research grants for Medtronic; Allison; Franҫois Mion is a consultant for Laborie and lecturer for Medtronic; P. Ronan O’Connell is a consultant for Medtronic; Satish Rao is a advisory board member for and grant research support from Medtronic and Intone MV; Medtronics Incorporated and provided research grant support for advisory board; Jose María Remes Troche is a member of Advisory Board for Takeda, Asofarma, and Astra Zeneca, received grants from Takeda, Sanfer, and Newton Foundation, is a speaker for Takeda, Asofarma, Sanfer, Sanofi Aventis, and Carnot; R. Matthew Reveille is a consultant for Diversatek Healthcare; S Mark Scott is a consultant and provided lectures and training courses for Laborie; Carolynne Vaizey provided research grants, and is a consultant, speaker, and educator for Medtronic Inc, and an educator for THD; Veronique Vitton is a consultant for Medtronic; William Whitehead provided research support from Medspira Instruments, Palette Life Sciences, and Allergan, and is a consultant for Ironwood, Takeda, and Valeant; Reuben Wong is a consultant for Laborie/MMS and Takeda.

## AUTHOR CONTRIBUTIONS

EVC led the organization of the group and the consensus voting, drafted the manuscript, organized the revision of the manuscript, and approved the final version. HH co‐organized group meetings and collated consensus voting, reviewed the manuscript, and approved the final version. CK, MF, and SMS acted as chairs of the group meetings reviewed the manuscript before circulation to the group as a whole, finalized the document with EVC, and approved the final version. SR acted as a chair of the group meetings and together with all other IAPWG members participated in the meetings, provided commentary on the manuscript, and approved the final version.
