# Supplementary material for: The international anorectal physiology working group (IAPWG) recommendations: Standardized testing protocol and the London classification for disorders of anorectal function
Source: Neurogastroenterol Motil. 2019 Aug 12;32(1):e13679. doi: 10.1111/nmo.13679 (PMC6923590; doi:10.1111/nmo.13679)
Supplement: Supplementary file 1 [file NMO-32-e13679-s001.docx]

**SUPPLEMENTARY TABLE 1**

| **IAPWG protocol sequence** | **Consensus level** |
| --- | --- |
| Stabilization duration of 3 minutes | C1 |
| Rest duration of 60 seconds | C2 |
| Short squeeze duration of 5 seconds | C1 |
| Number of short squeezes recommended = 3 | C1 |
| 30 second recovery interval between short squeezes | C1 |
| Long squeeze duration of 30 seconds | C1 |
| 60 second recovery interval after long squeeze | C1 |
| Number of coughs recommended = 2 | C1 |
| 30 second recovery interval between coughs | C1 |
| Number of pushes recommended = 3 | C1 |
| 30 second recovery interval between pushes | C1 |
| Rectal sensory testing | C1 |
| RAIR | C1 |

**SUPPLEMENTARY TABLE 2**

| **PART 1: DISORDER OF THE RECTOANAL INHIBITORY REFLEX** | |  |
| --- | --- | --- |
|  |  |  |
| **Element type** | **Element text** | **Consensus level** |
| Title | Disorder of the rectoanal inhibitory reflex | C1 |
| Decision point | RAIR not elicited | C1 |
| Diagnosis | Rectoanal areflexia | C1 |
| Clinical significance | Rectoanal areflexia = MAJOR | C1 |
| Negative study | No disorder of the rectoanal inhibitory reflex | C1 |
| Footnote | minimum volume required to elicit reflex not established in the literature: false negatives may be seen with low distending volumes in a large capacity rectum | C1 |
| Footnote | RAIR not elicited is a pattern not seen in health | C1 |
| Footnote | may indicate the need for further investigation to exclude aganglionosis especially in paediatric populations and adult patients with co-existent megarectum / megacolon | C1 |
|  |  |  |

| **PART 2: DISORDERS OF ANAL TONE AND CONTRACTILITY** | |  |
| --- | --- | --- |
|  |  |  |
| **Element type** | **Element text** | **Consensus level** |
| Title | Disorders of anal tone and contractility | C1 |
| Decision point | Anal resting pressure >ULN | C1 |
| Diagnosis | Anal hypertension | C1 |
| Clinical significance | Anal hypertension = MINOR | C1 |
| Decision point | Anal resting pressure <LLN | C1 |
| Decision point | Anal squeeze pressure <LLN | C1 |
| Diagnosis | Combined anal hypotension and hypocontractility | C1 |
| Clinical significance | Combined anal hypotension and hypocontractility = MAJOR | C1 |
| Diagnosis | Anal hypotension with normal contractility | C1 |
| Clinical significance | Anal hypotension with normal contractility = MAJOR | C1 |
| Decision point | Anal squeeze pressure <LLN | C1 |
| Diagnosis | Anal normotension with hypocontractility | C1 |
| Clinical significance | Anal normotension with hypocontractility = MAJOR | C2 |
| Negative study | No disorder of the anal tone or contractility | C1 |
| Footnote | The functional anal canal length may be measured, but its use as a diagnostic criterion in isolation is unproven | C1 |
| Footnote | May be associated with slow and/or ultraslow waves, however the clinical significance of these has not been established | C1 |
| Footnote | This finding may have greater clinical significance in certain patient groups (e.g. chronic anal fissure) | C1 |
| Footnote | Addition of an abnormal cough response may indicate a more severe phenotype but its use as a diagnostic criterion is unproven | C1 |
|  |  |  |

| **PART 3: DISORDERS OF ANORECTAL CO-ORDINATION** | |  |
| --- | --- | --- |
|  |  |  |
| **Element type** | **Element text** | **Consensus level** |
| Title | Disorders of rectoanal co-ordination | C1 |
| Decision point | Abnormal balloon expulsion test | C1 |
| Decision point | Rectal pressure change during push | C1 |
| Decision point | Anal pressure decrease (relaxation) during push >LLN | C2 |
| Diagnosis | Abnormal expulsion with normal pattern of rectoanal co-ordination | C1 |
| Clinical significance | Abnormal expulsion with normal pattern of rectoanal co-ordination = INCONCLUSIVE | C1 |
| Diagnosis | Abnormal expulsion with dyssynergia | C2 |
| Clinical significance | Abnormal expulsion with dyssynergia = MINOR | C3 |
| Decision point | Anal pressure decrease (relaxation) during push >LLN | C2 |
| Diagnosis | Abnormal expulsion with poor propulsion | C1 |
| Clinical significance | Abnormal expulsion with poor propulsion = MINOR | C3 |
| Diagnosis | Abnormal expulsion with poor propulsion and dyssynergia | C2 |
| Clinical significance | Abnormal expulsion with poor propulsion and dyssynergia | C2 |
| Decision point | Any abnormal pattern of anorectal co-ordination (as above) | C1 |
| Diagnosis | Normal expulsion with abnormal manometric pattern of rectoanal co-ordination | C1 |
| Clinical significance | Normal expulsion with abnormal manometric pattern of rectoanal co-ordination = INCONCLUSIVE | C1 |
| Negative study | No disorder of rectoanal co-ordination | C1 |
| Footnote | Impaired evacuation of contrast medium on alternative testing e.g. barium or MR defaecography | C1 |
|  |  |  |

| **PART 4: DISORDERS OF RECTAL SENSATION TO DISTENSION** | |  |
| --- | --- | --- |
|  |  |  |
| **Element type** | **Element text** | **Consensus level** |
| Title | Disorders of rectal sensation | C1 |
| Decision point | 2/3 sensory parameters >ULN | C1 |
| Diagnosis | Rectal hyposensitivity | C1 |
| Clinical significance | Rectal hyposensitivity = MAJOR | C1 |
| Decision point | 1/3 sensory parameter >ULN | C1 |
| Diagnosis | Borderline rectal hyposensitivity | C1 |
| Clinical significance | Borderline rectal hyposensitivity = INCONCLUSIVE | C1 |
| Decision point | 1 or more sensory parameter(s) <LLN (to include MTV) | C1 |
| Diagnosis | Rectal hypersensitivity | C1 |
| Clinical significance | Rectal hypersensitivity = MAJOR | C1 |
| Negative study | No disorder of rectal sensation to distension | C1 |
| Footnote | abnormal results may be further described using additional methods (e.g. barostat to assess compliance) | C1 |
